# The Use of the Fluorometer in Röntgen Ray Work1Read at the thirtieth annual meeting of the Medical Association of Central New York, held at Buffalo, October 19, 1897.

**Published:** 1898-04

**Authors:** John Dennis

**Affiliations:** Rochester, N. Y.


### THE USE OF THE FLUOROMETER IN RÖNTGEN RAY WORK.[1]

By JOHN DENNIS, Rochester, N. Y.

DURING the brief minutes allotted to this paper, it would obviously be impossible to enter upon a disquisition on the Röntgen energy, if, indeed, it would not be a work of supererogation. You are all familiar with the general principles of this strange force, in so far as they have been demonstrated. The effort, therefore, will be to confine consideration to personal researches on the lines of using the energy as an aid to surgeons and physicians.

Every devotee of the Röntgen energy who desires to utilise it in surgery is confronted with some very serious and not always fully understood conditions; conditions which must be recognised and rectified before the force can be relied upon as a guide in the search for the normally invisible in the human organism.

The effect of the force becomes manifest to the observer in the form of a shadow, whether it be the manifestation on the screen

---

1. Read at the thirtieth annual meeting of the Medical Association of Central New York, held at Buffalo, October 19, 1897.

of the fluoroscope, or the result of action on a sensitive, photographic plate; it is a shadow and a shadow only. This shadow is subject to very grave distortions. In the first place, the slightest variation in the position of the subject, with reference to the source of energy, produces a grotesque effect in the shadow of the subject itself. The grotesque effect in the shadow of any impermeable object inside of the subject is still more marked. Take an orange and place a bit of metal just inside, at say the stem end, then hold the orange firmly against the screen of the fluoroscope before the tube, change your position laterally, ever so little, and you will observe that metal travel round the end of the orange in a manner which is uncanny. You will not at first believe the evidence of your own eyes.

There is, then, a proper position in which to place a subject in order to produce a correct shadow. Having accomplished this result, the operator is still in trouble. The rays constitute true radii, with the point of convergence at the center of the anode of a focus tube. From this point the lines of force proceed straight toward infinity. The first authentic instance of deflecting the force by means of a magnet is yet to be noted, although a magnet will affect the cathode rays in the tube. Such being the case, there is the further distortion caused by the radiation of the lines of force.

To rectify this distortion of position and the distortion caused by the radiation of the force, in order that surgeons and physicians might be able to form correct conclusions in Röntgen work, was the problem which presented itself in devising the instrument called the fluorometer. Time will not admit of entering into all the details of the labors in this behalf. You are asked to give credit for candor in the statement of results, which have been fully verified in private practice and hospital practice, as well as in the laboratory.

By means of the appliance, which has been called for convenience the fluorometer, the physician or surgeon or any other experimenter in the field of the Röntgen energy is enabled to accomplish these results unerringly:

*First*—To produce a correct shadow of the subject on the field of the fluoroscope, and to reproduce the effect there observed on the sensitive plate, if desired.

*Second*—To reproduce on the operating table exactly the position occupied by the subject when the observation and fluorograph were taken.

*Third*—To furnish an accurate cross-section of the subject, with the foreign object or any desired part of the anatomy in close juxtaposition.

*Fourth*—To fix with exactness the line through that cross-section upon which any foreign substance in the human organism, which is observable on the fluoroscope, is situated and by means of markings on the surface establish that line for future exploration.

*Fifth*—To establish a second line, at right angles to the first, at the intersection of which two lines the foreign substance is located, with mathematical exactness; that is, for instance, a cross-section of the chest is produced, exactly as if the body were severed at the point desired. Then this "end" is divided by straight lines, at the intersection of which is the more or less impermeable object sought.

*Sixth*—An undistorted view of any portion of the bones of the skeleton is obtained, which can be manifested in the Röntgen shadow. If it be desired to observe a case of congenital dislocation of the hip in a child, for instance, a cross-section of the child's body is obtained and the observer is furnished also with a vertical line passing through the cross-section at any or all points desired.

In brief, gentlemen, the endeavor is to furnish a certain and unerring guide to your observations of the interior of the human organism, so far as disclosed in the present state of the art, and supply a chart for exploration, which will enable surgeons to proceed with absolute certainty.

Without trespassing in the least on the field of the surgeon or physician, it is the function of the fluorometer to furnish absolute data regarding an object which is more impermeable than its surroundings or other objects, and to indicate its precise situation. The object sought may be a calculus, a bullet, a needle, a bit of glass or a coin in the alimentary canal; or it may be desired to present an undistorted view of a fracture, a dislocation or a diseased bone. That accomplished, the appliance has fulfilled its mission. The fluorometer has no knife, forceps, probe or other surgical attachment. If success has been achieved in furnishing an appliance by which the surgeon can apply his skill to better advantage, if it enables him to more surely avail himself of this wonderful and, indeed, almost supernatural force in the alleviation of human suffering and the saving of human life, then the long and somewhat arduous experiments will not have been in vain.

The object of the endeavor has been to make certain that which is uncertain and to furnish means for unerring diagnosis, so far as diagnosis can be made by means of the Röntgen energy.

